# Elevation, Temperature, and Aquatic Connectivity All Influence the Infection Dynamics of the Amphibian Chytrid Fungus in Adult Frogs 

**DOI:** 10.1371/journal.pone.0082425

**Published:** 2013-12-04

**Authors:** Sarah J. Sapsford, Ross A. Alford, Lin Schwarzkopf

**Affiliations:** School of Marine and Tropical Biology, James Cook University, Townsville, Queensland, Australia; University of Georgia, United States of America

## Abstract

Infectious diseases can cause population declines and even extinctions. The amphibian chytrid fungus, *Batrachochytrium dendrobatidis* (*Bd*), has caused population declines and extinctions in amphibians on most continents. In the tropics, research on the dynamics of this disease has focused on amphibian populations in mountainous areas. In most of these areas, high and low elevation sites are connected by an assemblage of streams that may transport the infectious stage of the pathogen from high to low elevations, and, also, this pathogen, which grows well at cool temperatures, may persist better in cooler water flowing from high elevations. Thus, the dynamics of disease at low elevation sites without aquatic connections to higher elevation sites, i.e., non-contiguous low elevation sites, may differ from dynamics at contiguous low elevation sites. We sampled adult common mistfrogs (*Litoria rheocola*) at six sites of three types: two at high (> 400m) elevations, two at low elevations contiguous with high elevation streams, and two at low elevations non-contiguous with any high elevation site. Adults were swabbed for *Bd* diagnosis from June 2010 to June 2011 in each season, over a total of five sampling periods. The prevalence of *Bd* fluctuated seasonally and was highest in winter across all site types. Site type significantly affected seasonal patterns of prevalence of *Bd*. Prevalence remained well above zero throughout the year at the high elevation sites. Prevalence declined to lower levels in contiguous low sites, and reached near-zero at non-contiguous low sites. Patterns of air temperature fluctuation were very similar at both the low elevation site types, suggesting that differences in water connectivity to high sites may have affected the seasonal dynamics of *Bd* prevalence between contiguous and non-contiguous low elevation site types. Our results also suggest that reservoir hosts may be important in the persistence of disease at low elevations.

## Introduction

 Emerging infectious diseases (EIDs) can pose major threats to wildlife species [1]. At the individual level, disease can cause illness and may cause death, and at the population level, disease can cause declines and extinctions [2-6]. Infection prevalence, rates of transmission to susceptible hosts, and pathogen load within hosts are the driving forces behind the dynamics of infectious diseases [7,8]. It is unusual for diseases to cause extinctions, because population declines caused by pathogens are typically self-limiting, in that, as pathogens cause host populations to decline, the populations reach low levels at which transmission does not exceed loss and the pathogen dies out [1]. Extinctions are possible, however, when a pathogen has a resting stage, or when a pathogen persists in the environment by other means, or has a reservoir host or hosts [1]. Reservoir hosts are typically less susceptible species that can sustain the pathogen, allowing it to persist in the environment even when susceptible species decline and disappear from the system [4,9-11]. Determining the dynamics of diseases is important in understanding their effects on host populations, and also in providing possible ways of mitigating host declines (e.g., by reducing transmission rates).

 Chytridiomycosis, caused by the amphibian chytrid fungus, *Batrachochytrium dendrobatidis* (*Bd*), has caused many population declines, and both local and global extinctions of frogs in many parts of the world [5,12,13]. *Bd* infects species over large geographic areas, even in areas that are not highly disturbed by humans; the greatest numbers of chytridiomycosis-caused declines have occurred in stream-associated species [13-15]. The fungus is transmitted via motile aquatic zoospores [16]. 

 The growth of *Bd* in culture is temperature dependent [17]: an isolate from the Australian Wet Tropics grew optimally between 15°C and 25°C, with growth slowing dramatically from 26°C to 28°C; the fungus dies above 30°C [17]. Thus, the prevalence of this pathogen can be affected by environmental temperatures [13,15,18-21], and, accordingly, the air temperature to which individual frogs are exposed has an effect on their disease status [21,22] (E. A. Roznik, unpublished data). Also, at high elevation sites, air temperatures are cooler, on average, than at low elevations [15,22], and in Australia, *Bd* prevalence tends to be higher at high elevations, which we define as those above 400 m, for the approximate elevational cut-off above which chytridiomycosis caused severe frog population declines in the Australian Wet Tropics [14,22]. Prevalence of *Bd* is also higher during colder, winter months than in warmer, summer months [13,15,19,20]; this seasonal pattern occurs at both high and low elevations [15,20]. 

 High and low elevation areas are often connected by streams, making them contiguous, such that water flows from high to low elevation sites. In such streams, *Bd* zoospores may be carried from high to low elevations, influencing the prevalence of *Bd* in the populations at lower elevations through drift [23]. In addition, cooler water flows from high elevations to low elevations, potentially reducing water temperatures in low elevation streams that flow from high elevations [24]. Cooler water temperatures and the flow of infectious propagules may affect infection dynamics in aquatic larvae, and could also affect disease dynamics in adult frogs of species that frequently come into contact with water [24]. Not all low elevation sites, however, are connected to high elevation sites. Some low elevation streams have no adjacent regions higher than 400 m. Differences in drift of pathogen propagules, and the degree of moderation of maximum temperatures could cause low areas non-contiguous with high elevation streams to have different infection dynamics than low areas that are contiguous with high elevation streams. 

 The aim of our study was to quantify the infection dynamics of *Bd* in one frog host species across high elevation, contiguous low elevation, and non-contiguous low elevation sites, and to determine how those dynamics differ with air temperature and among all three types of sites. We surveyed six populations of the adult common mistfrog, *Litoria rheocola*, two at each site type (i.e., at all possible combinations of elevation and aquatic connectivity), over a one-year period from midwinter to the following midwinter. This is the first comprehensive study of the seasonal dynamics of the amphibian chytrid fungus in multiple populations of adult frogs to incorporate replicate sites in uplands and lowlands, and to examine the importance of connectivity of water flow to disease dynamics. 

## Materials and Methods

### Ethics statement

 The project was carried out under permit WITK03070508 issued by the Queensland Department of Environment and Resource Management, and we adhered to animal ethics protocols approved by the James Cook University Animal Ethics Committee (approval A1420).

### Study Species

 The adult common mistfrog (*Litoria rheocola*) is a small (mean male body size: 1.98 ±0.15 g SD, 30.3 ± 1.16 mm SD snout-urostyle length; mean female body size: 2.78 ± 0.59 g SD, 34.83 ± 3.06 mm SD snout-urostyle length), endangered, hylid frog [25]. *Litoria rheocola* declined and disappeared from high elevation sites in the Australian Wet Tropics bioregion in the 1990s [1,26,27], but has since reappeared at some high elevation sites [14,27]. It occurs in rocky, fast-flowing, rainforest streams in northern Queensland, Australia [28,29]. Little is known about the natural history of *L. rheocola*; however, at night, and on rainy days, individuals typically perch on rocks, logs, and stream-side vegetation near riffles [28-30]; on dry days they typically shelter between moist rocks in the stream bed (E.A. Roznik, unpublished data). 

### Study Sites

 Sampling of adults took place over one full annual cycle, from austral winter (June/July 2010), through spring (October 2010), summer (January 2011), autumn (March/April 2011) and the following winter (June/July 2011). Adults were sampled at six sites in the Wet Tropics Bioregion in northern Queensland, Australia. The sites were of three types: two were at high (>400 m) elevations (“High” sites; Table 1), two were at low elevations contiguous with (and downstream from) other high elevation sites (“Contiguous low” sites; Table 1), and two were at low elevations that were not downstream from high elevation sites (“Non-contiguous” sites; Table 1). All sampled creeks were independent from each other; although the low elevation sites were contiguous with higher elevation sites, those were not the high elevation sites we studied; there was no connection via flow between our high and low elevation sites. All of the creeks were surrounded by tropical rainforest and all contained pools and riffles; some also had waterfalls. All creeks shared similar characteristics, although the composition of the creek beds varied somewhat among sites: Frenchman Creek, Tully Creek, and Bobbin Bobbin Falls were comprised of large boulders interspersed with sections of small rocks (1-10 cm in diameter). In comparison, the beds of Windin, Mena, and Stoney Creeks were primarily comprised of small rocks. Frenchman Creek had the greatest width, at approximately 10 m, whereas the other creeks were 3 - 4 m wide. All creeks had sections that varied in flow rates (0.5 m/s - 2 m/s): in all creeks some sections (runs, riffles, and waterfalls) flowed at high rates whereas pool areas had much lower flow rates. Vegetation was similar at and around all creeks with a canopy of rainforest trees largely covering the streams. Understory vegetation included vines, ferns, and palms.

**Table 1:** Sites at which samples were taken within the Wet Tropics Bioregion in northern Queensland, Australia.

Site	National Park	Site Type	Location	Elevation (m)
Windin Creek	Wooroonooran	High	17°22’04.2”S 145°42’52.1”E	718
Bobbin Bobbin Falls	Wooroonooran	High	17°22’43.5”S 145°46’21.7”E	700
Frenchman Creek	Wooroonooran	Contiguous low	17°18’29.2”S 145°55’16.2”E	59
Tully Creek	Tully Gorge	Contiguous low	17°46’29.5”S 145°38’38.2”E	114
Mena Creek	Private land	Non-contiguous	17°38’59.6”S 145°59’13.5”E	59
Stoney Creek	Hull River	Non-contiguous	17°55’17.9”S 146°4’7.2”E	18

### Field Methods

 Prior to surveys, we placed flags at 10 m intervals along a 400 m transect at each site so we could record the location of each frog that was captured. Sampling was conducted over five nights at each site in each season. Adult frogs were located at night using visual and auditory cues and were captured by hand, using clean plastic sandwich bags worn as a glove. Following capture, all frogs were swabbed for *Bd*, sexed (using presence/absence of nuptial pads), measured to the nearest 0.1 mm (snout-urostyle length), weighed to the nearest 0.1 g, and individually marked with visible implant elastomer (VIE; to distinguish recaptured individuals). To prevent disease transmission among frogs, a new bag was used to capture each frog and a new pair of latex gloves was used to handle each individual. In addition, all equipment and shoes were sterilized when travelling between different sites to prevent disease transmission. Once processing was complete, frogs were released at their capture location. 

### Assessing disease status

 Frogs were swabbed using a sterile, fine-tip, dry rayon swab (#113, Dry swabs, Medical Wire and Equipment, Corsham, Wiltshire U.K.). The ventral side of each foot, inner thigh area, both lateral sides of the stomach, medial section of the stomach, and ventral side of each hand were swabbed three times because the fungus is mainly found on these areas of the body [31,32].

 After processing, all swabs were placed in separate, labelled vials and refrigerated until the end of sampling. All swab samples were processed using real-time quantitative PCR [33]. Samples were run in triplicate and considered positive for *Bd* if at least two of the three PCR reactions had numbers of zoospore equivalents that were greater than zero.

### Environmental temperatures

 Air temperature was recorded using three Thermochron iButton dataloggers (model DS1921G, precision: 0.5 °C, accuracy: 1°C) at each site. They were placed in shaded locations at 0 m, 200 m, and 400 m along the transect, approximately 1 m from the creek edge and 2 m above the ground. These dataloggers were coated in transparent plastic (Plasti Dip, Plasti Dip International, Blaine, MN, USA) to prevent water damage [34]. Each iButton recorded air temperature at 90 min intervals over the entire study period (June 2010 to June 2011). 

### Analysis of prevalence and individual probability of infection

 The great majority (1676 of 1866) of records of infection status we obtained during the study were from adult males. We initially examined overall patterns of prevalence of infection by class of individual (male, female, juvenile); that comparison indicated that patterns differed among classes, but we had relatively little data for females and juveniles. We therefore carried out all of our more detailed analyses using adult males only. 

We initially examined the overall relationship between mean prevalence of infection in adult male frogs at each site and air temperature using a linear regression. Because prevalence is a proportion, and is non-normally distributed, we arcsine square-root transformed it before analysis. The mean of the air temperatures we measured at each site during each sampling trip (seasonal mean air temperature) was used as the independent variable in the linear regression. 

 To determine in detail whether there were effects of season, site type (combination of elevation and aquatic connectivity), and their interaction [15,35] in addition to the overall seasonal effects of temperature, we built generalized linear mixed models with a binomial link function using the glmer function in R version 2.15 [36]. Individual disease status (infected or not infected) was the response variable. We used the two sites at each site type (high, contiguous low, non-contiguous low) as replicates; all models included site as a random effect, to account for site-specific differences in the probability of infection that were not related to the effects of interest. Due to significant damage caused by Tropical Cyclone Yasi (category 5), which hit the north-eastern Queensland coast in February 2011, one of the high sites (Bobbin Bobbin Falls) was not accessible in autumn 2011. We therefore excluded data for the autumn season from our models. We started with a model that included only the random effect of site and an intercept. We then added seasonal mean air temperature as a covariate, and tested the significance of the increased fit against that of the intercept-only model using Wald’s Χ^2^ in the ANOVA function in R. We then added effects of season, site type, and their interaction in that order; with the addition of each effect, we tested the significance of the new model as compared to the preceding model using the ANOVA function. When the site type X season interaction was significant, we examined the effects of sites in greater detail by running a series of post-hoc analyses on subsets of the data containing only data from two site types; in each subset analysis, the full model containing the interaction was compared to a reduced model containing all other effects including the covariate and the random effect of site.

### Analysis of intensity of infection

 We analyzed data on the intensity of infection of individuals using a series of generalized linear mixed models with an identity link function using the glmer function in R version 2.15 [36]. These analyses included only data from infected males. *Bd* zoospore equivalents were log_10_ transformed prior to analysis. Modelling and hypothesis testing followed the procedures outlined above for individual probability of infection.

## Results

 We determined the infection status of 1866 frogs. These results appear by class of individual in Table 2. Overall, the probability of infection differed significantly among classes of individuals (Table 2). Females were more likely to be infected than males (27.2% versus 20.1%, overall). Juveniles were far more likely to be infected than adults of either class (42.9% overall); this may in part reflect the fact that most juveniles were encountered in winter when the probability of infection was higher in males (see below). Excluding the metamorph class because of low sample size, the prevalence of infection differed significantly among classes of individuals (Fisher’s Exact test, P < 0.0001).The great majority of individuals for which we obtained infection status (1676 individuals) were adult males. They were reasonably evenly distributed among sites and seasons (winter (N = 354), spring (N = 535), summer (N = 314), autumn (N = 363) and winter the following year (N = 110)), and were the only class of individuals with sufficient data for more detailed analysis. 

**Table 2:** Infection status of individuals of different classes in northern Queensland, Australia.

Class	Number infected	Number uninfected	Total count
Female	31	83	114
Male	338	1338	1676
Subadult	29	22	51
Juvenile	9	12	21
Metamorph	3	1	4

Numbers are aggregated across all populations and sampling dates. Excluding the metamorph class because of low sample size, the prevalence of infection differed significantly among classes of individuals (Fisher’s Exact test, P < 0.0001).

### Temperature

**Figure 1:**
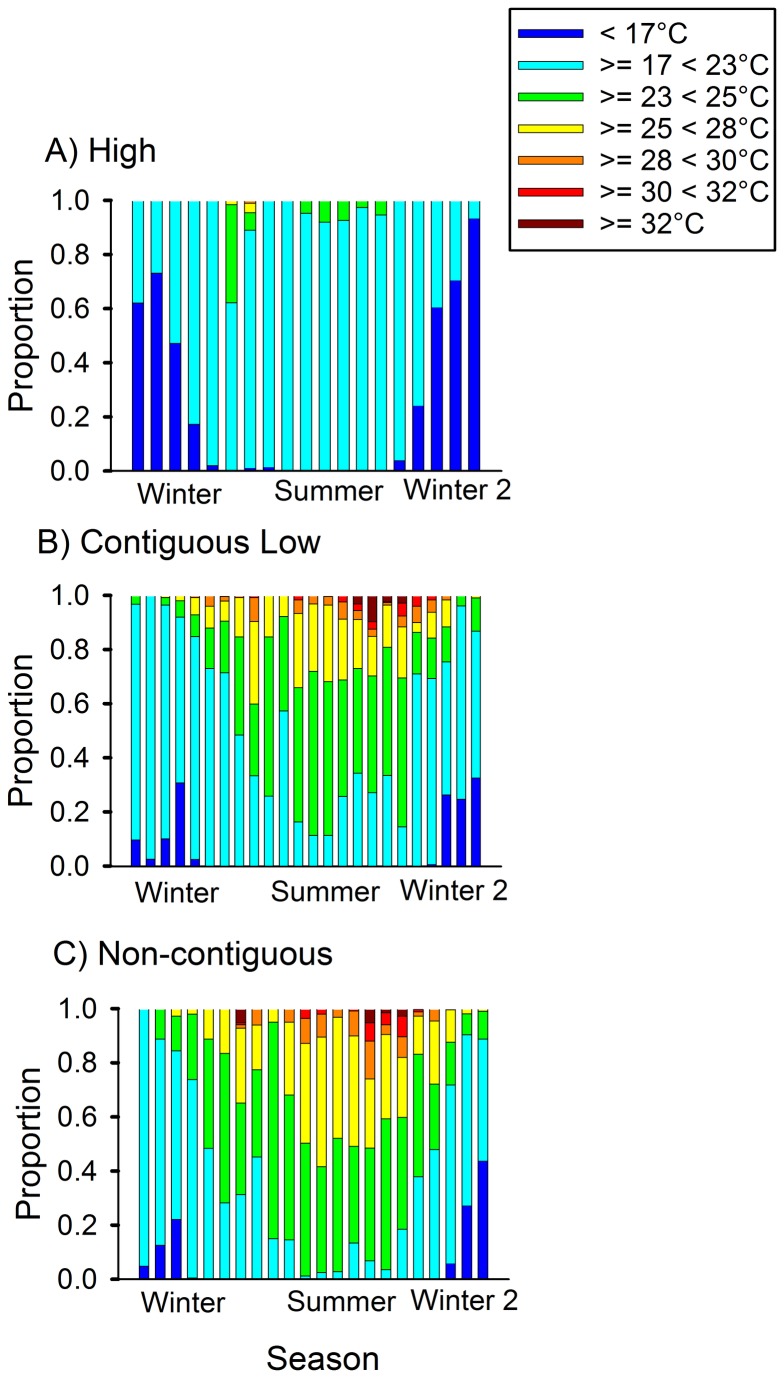
Proportion of environmental air temperatures in ranges relevant to *Batrachochytrium dendrobatidis* growth at one high site, one contiguous low site and one non-contiguous low site. Temperatures were measured at two high elevation rainforest sites (Bobbin Bobbin Falls and Windin Creek), two contiguous low elevation rainforest sites (downstream from high elevation; Frenchman and Tully Creek), and two non-contiguous low elevation rainforest sites (not downstream from high elevation; Mena and Stoney Creek) in northern Queensland, Australia. *Batrachochytrium dendrobatidis* growth occurs between 4°C and 25°C, optimal growth between 17°C and 25°C, slow growth less than 10°C and above 25°C, and dies at 30°C and above. Each bar represents the proportion of air temperature in each range of *Bd* temperature during five two week periods from June 2011 to June 2012. These coincide with our sampling periods; because they include two winter periods, the proportions of time at lower temperatures are probably over-represented compared to a simple annual cycle.

 We determined the proportion of air temperatures measured at each site that fell within a series of ranges relevant to *Bd* growth. The proportions differed among site types (combination of elevation and aquatic connectivity), but the sites within each type had similar temperature patterns (Figure 1). The high sites were much cooler than both the contiguous and non-contiguous low sites, even in the summer months. Air temperatures at the high sites were typically either less than 17°C (20.9%), which might slow *Bd* growth slightly, or between 17°C and 23°C (74.5%), which is within the range for optimal *Bd* growth [17,21]. Air temperatures at the high sites were never above 28°C, and were only between 23°C and 28°C 4.5% of the time. Air temperatures at both types of low elevation sites were similar to one another, and substantially higher than those at high elevation sites: low elevation sites spent more time between 23°C and 28°C (38.8%) and during summer, temperatures were frequently above 25°C, which substantially slows *Bd* growth, and were sometimes above 30°C (1.2%), which is within the range that kills *Bd* [17]. 

### Temperature and prevalence

**Figure 2:**
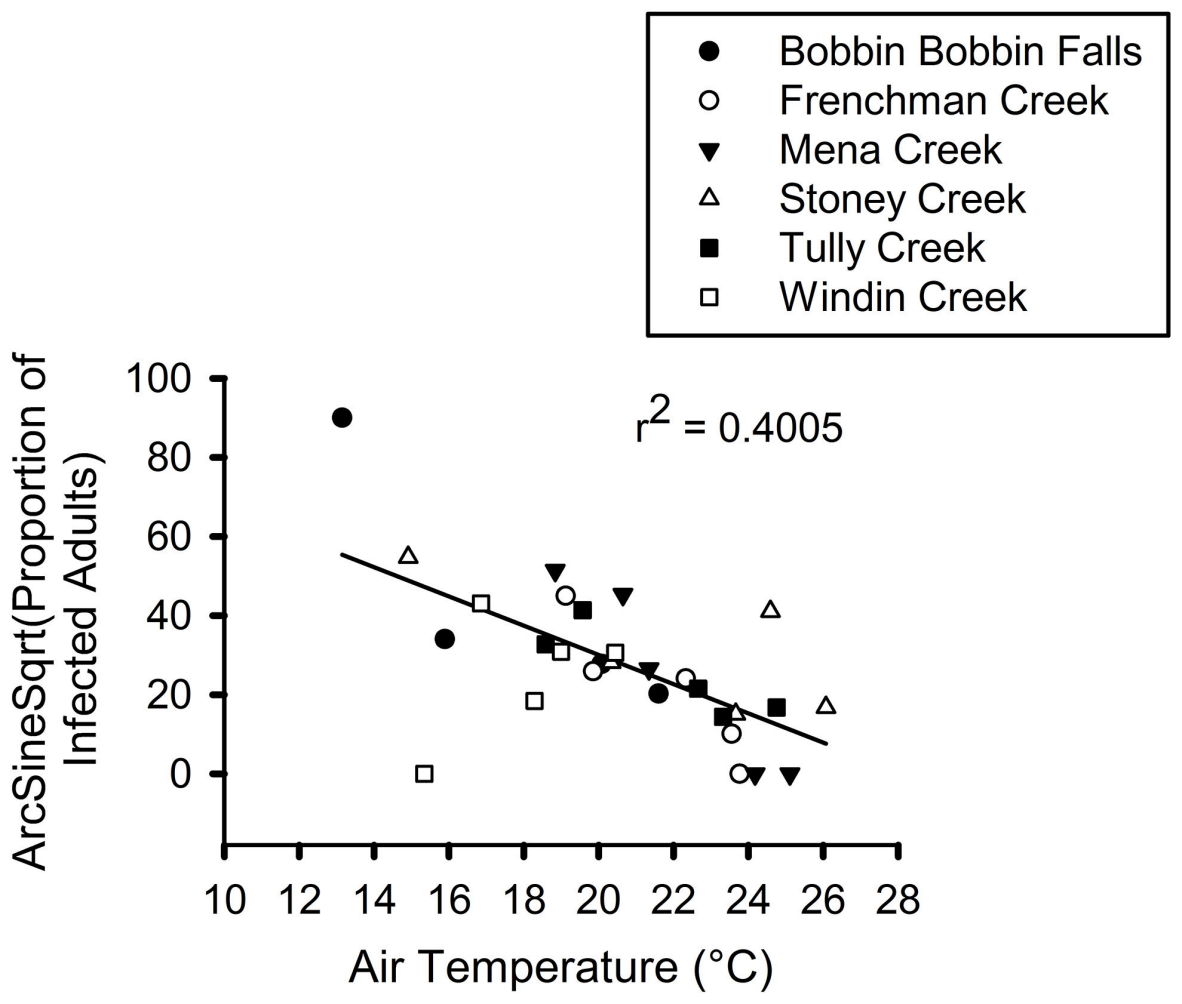
Relationship between air temperature and prevalence of *Batrachochytrium dendrobatidis*. Temperatures and prevalence were measured (for which data were available) at two high elevation rainforest sites (Bobbin Bobbin Falls and Windin Creek), two contiguous low elevation rainforest sites (downstream from high elevation; Frenchman and Tully Creek), and two non-contiguous low elevation rainforest sites (not downstream from high elevation; Mena and Stoney Creek) in northern Queensland, Australia at each season frogs were sampled.

 Across all combinations of site and season, there was a significant negative correlation between seasonal mean air temperature at sites and *Bd* prevalence in adult male frogs (r^2^ = 0.4005; n = 29; P = 0.0002; Figure 2) indicating that prevalence was significantly lower at higher air temperatures. 

### Site type, infection status, and probability of infection

**Figure 3:**
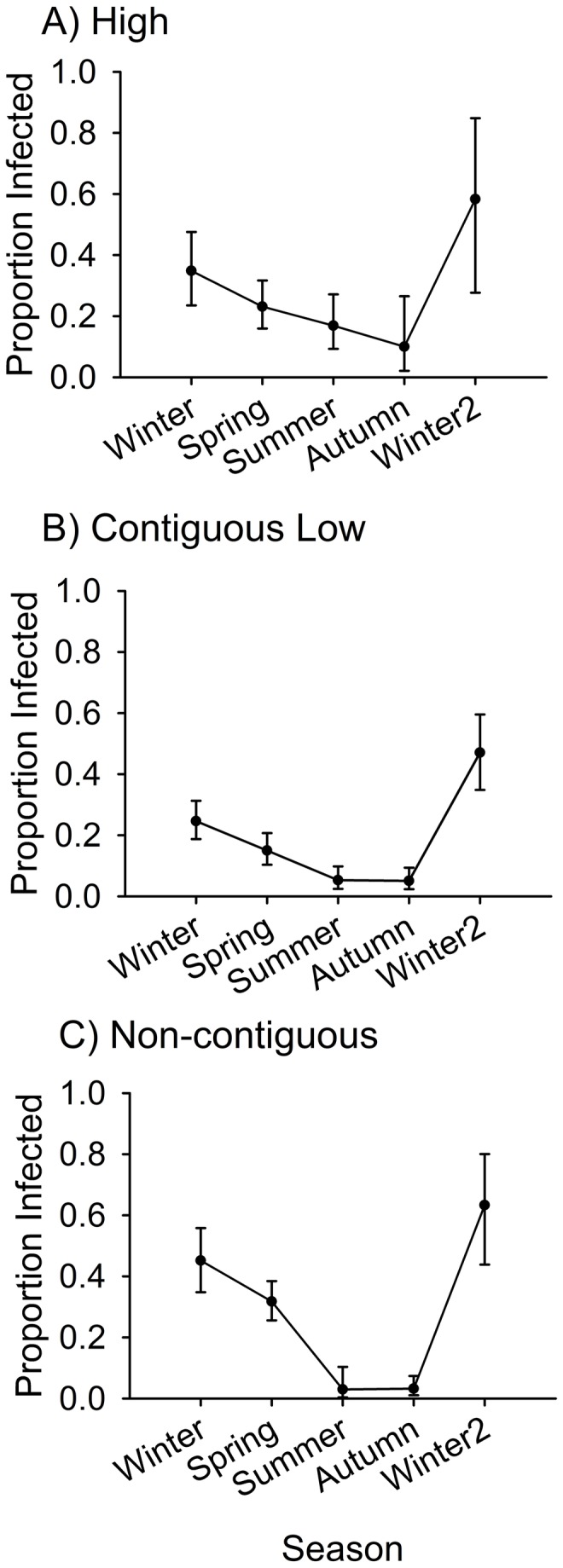
Prevalence of *Batrachochytrium dendrobatidis* of adult frogs at different site types in northern Queensland, Australia with 95% confidence intervals. Prevalence was calculated for each season from Winter 2010 to Winter 2011 at two high elevation rainforest sites (Bobbin Bobbin Falls and Windin Creek), two contiguous low elevation rainforest sites (downstream from high elevation; Frenchman and Tully Creek), and two non-contiguous low elevation rainforest sites (not downstream from high elevation; Mena and Stoney Creek).

 In addition to the significant effects of seasonal mean air temperature, there were other effects on the probability of infection of individual male frogs (Table 3). Infection probability was also significantly affected by season, site type (i.e., the combination of elevation and aquatic connectivity) and the season X site type interaction. The interaction between season X site type for data at high and contiguous low sites was not significant, indicating that the effects of season on infection probability did not differ significantly between high and contiguous low sites. The post-hoc analysis including data only for contiguous and non-contiguous low sites also showed no significant difference in effects of season among sites (Table 3). However, a test for models containing data from high elevation sites and non-contiguous sites (Table 3) demonstrated that the site type X season interaction was significant, indicating that the effect of season in non-contiguous low sites was different from that in high sites. Examining prevalence by site type and season (Figure 3) revealed that there were differences among site types; prevalence never approached zero in high elevation sites, whereas prevalence in non-contiguous low sites came very close to zero during summer and autumn, and was somewhat higher than at high elevation sites in winter and spring. The pattern at contiguous low sites was intermediate; prevalence at contiguous low sites was always lower than high sites, but was higher than non-contiguous sites in summer and autumn. These results show that although air temperature is important in determining the effects of sites and seasons on prevalence of *Bd*, there were additional effects not accounted for by temperature.

**Table 3:** Results of ANOVA comparisons of increasingly complex generalised linear mixed models for infection status of individual male frogs, using a binomial link function.

Effect			Wald chi-square	d.f.	P
Full data set					
Temperature			76.13	1	<0.0001
Season			43.39	3	<0.0001
Site type			11.98	2	0.0025
Season X Site type			13.07	6	0.0419
Tests of the season X site type interaction in two-site-type subsets					
High and contiguous low			3.90	3	0.2721
Contiguous low and non-contiguous			5.83	3	0.1199
High and non-contiguous			13.13	3	0.0044

All models included the random effects of site in addition to the specified effects. We initially compared a model including only the effect of seasonal mean air temperature at sites to an intercept-only model. We then added the effect of season, then the effect of site type (combination of elevation and habitat connectivity), and finally the site type X season interaction, in each case comparing the model to the preceding model. We next carried out three comparisons of pairs of models to determine which site types were responsible for the significant interaction. We created data sets containing only data from pairs of site types, and tested for the significance of the interaction in each data set; we Bonferroni-adjusted these results by setting alpha = 0.0167.

### Intensity of infection

**Figure 4:**
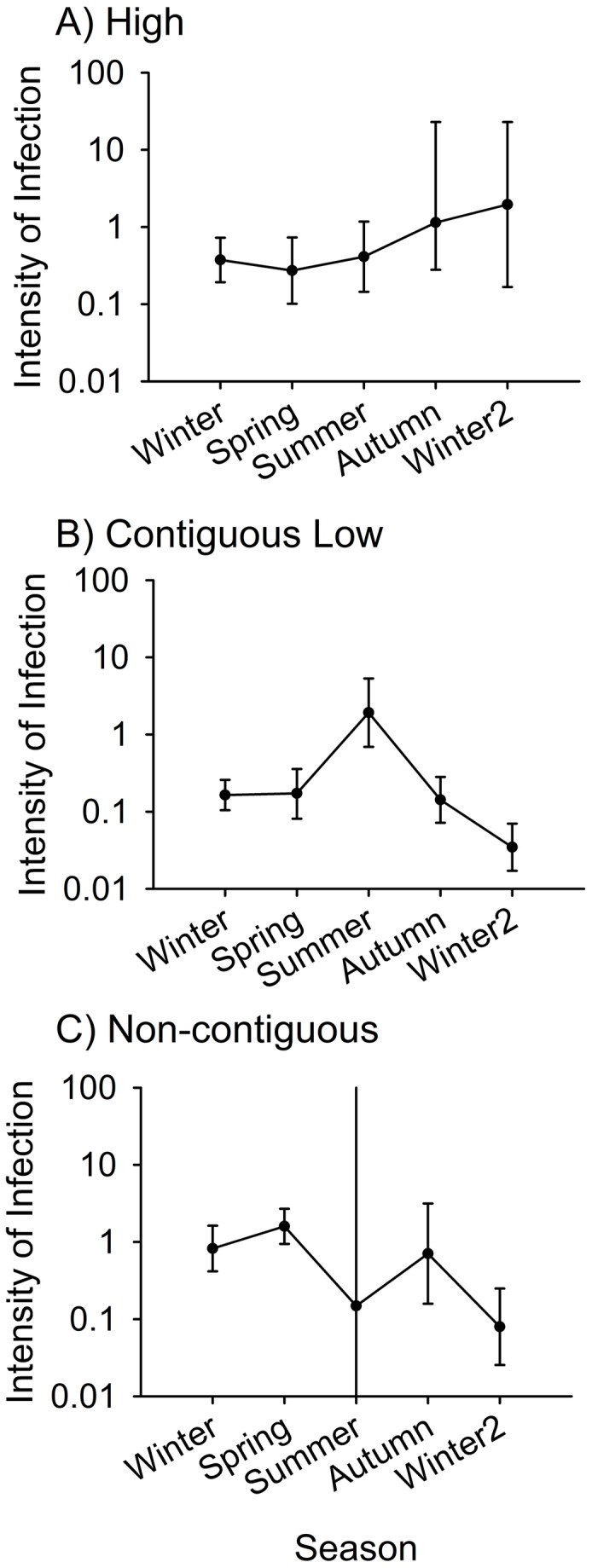
Intensity of infection of *Bactrachochytrium dendrobatidis* of adult frogs at different site types in northern Queensland, Australia with 95% confidence intervals. Intensity of infection was collected for each frog for each season from Winter 2010 to Winter 2011 at two high elevation rainforest sites (Bobbin Bobbin Falls and Windin Creek), two contiguous low elevation rainforest sites (downstream from high elevation; Frenchman and Tully Creek), and two non-contiguous low elevation rainforest sites (not downstream from high elevation; Mena and Stoney Creek). Confidence intervals for summer at non-contiguous sites have been truncated as they are very large due to small sample size (n=1).

 Intensity of infection was significantly influenced by temperature, season, site type, and their interaction (Table 4, Figure 4). The interaction of site type X season in high elevation and contiguous low sites was not significant following Bonferroni adjustment for multiple comparisons, although the non-adjusted P value suggested that the effects of season on infection probability may have differed between high and contiguous low sites in our data. Tests for models using data from contiguous low and non-contiguous low sites, and data from high and non-contiguous low sites indicated that the effects of season on site type differed significantly only between the high and non-contiguous low site types (Table 4). Examination of seasonal patterns for each site type (Figure 4) indicated that mean intensity of infections remained relatively constant over the year of the study at high elevation sites. Mean intensity of infections fluctuated seasonally in patterns that were superficially similar, except for summer, at contiguous and non-contiguous low sites; intensity peaked in summer at contiguous sites and was lower in summer at non-contiguous sites. The intensity of infection was substantially higher in frogs at non-contiguous low sites in summer in comparison to the other seasons. The very low intensity of infections at non-contiguous low sites in summer was determined from a very small sample and thus had very broad 95% confidence limits (Figure 4); this was because prevalence of infection was extremely low at these sites in summer. 

**Table 4:** Results of ANOVA comparisons of increasingly complex generalised linear mixed models for intensity of infection of infected male frogs only, using an identity link function.

Effect		Wald chi-square	d.f.	P
Full data set				
Temperature		10.11	1	0.0015
Season		14.91	3	0.0019
Site type		11.46	2	0.0033
Season X Site type		29.74	6	<0.0001
Tests of the season X site type interaction in two-site-type subsets				
High and contiguous low		9.24	3	0.0263
Contiguous low and non-contiguous		6.76	3	0.0800
High and non-contiguous		20.74	3	0.0001

All models included the random effects of site in addition to the specified effects. We initially compared a model including only the effect of seasonal mean air temperature at sites to an intercept-only model. We then added the effect of season, then the effect of site type (combination of elevation and habitat connectivity), and finally the site type X season interaction, in each case comparing the model to the preceding model. We next carried out three comparisons of pairs of models to determine which site types were responsible for the significant interaction. We created data sets containing only data from pairs of site types, and tested for the significance of the interaction in each data set; we Bonferroni-adjusted these results by setting alpha = 0.0167.

## Discussion

 Our results demonstrated, for the first time to our knowledge, that connectivity between high and low elevation sites may influence the seasonal patterns of prevalence of *Bd* in frog populations, such that sites connected to high elevation sites by water flow (our contiguous sites) had higher prevalence of disease in adult frogs. We also demonstrated that the dynamics of *Bd* infections on *L. rheocola* were influenced by temperature and season, effects that have been reported in previous studies on several species in Australia and elsewhere [15,18-20,35-37]. Prevalence of *Bd* in adult frogs was higher in cooler than in warmer months, and was higher at higher elevations, as has been reported elsewhere [18,38,39]. It seems likely that a substantial proportion, but not all, of the seasonal and elevational effects were caused by environmental temperature differences. We found that at low elevation sites, but not high elevation sites, summer air temperatures reached levels that should eliminate *Bd* infections from frogs (Figure 1). The prevalence of infection approached zero in populations in both contiguous and non-contiguous low sites in summer and autumn (Figure 3). Prevalence in populations at high elevation sites, by comparison, remained well above zero throughout summer and autumn. *Bd* grows and reproduces well within the range of thermal regimes of individual *L. rheocola* frogs, air, and water temperatures recorded at sites in northern Queensland [40]. 

 The seasonal pattern of prevalence did not differ significantly between high and contiguous low sites, or between contiguous and non-contiguous low sites, but did differ significantly between high and non-contiguous low sites. Prevalence in summer and autumn at contiguous low sites was intermediate between high and non-contiguous low sites (Figure 3), although air temperature patterns at the two types of low elevation sites were very similar (Figure 1). This suggests that the prevalence of *Bd* infections in adult populations at contiguous low sites may be affected by (i) drift of zoospores, or (ii) by the influx of cooler water from high elevation sites (average water temperature at high elevation sites: 18.4°C, contiguous low elevation sites: 20.6°C, and non-contiguous low elevations sites: 22.0°C; [24]), or both, at least during summer and autumn, coupling their disease dynamics to adjacent high elevation sites. In contrast, at non-contiguous low sites, prevalence approached zero in summer and autumn, but was slightly greater than at sites of either of the other two types in winter. The greater seasonal fluctuations in prevalence evident at non-contiguous sites may reflect their higher range of water temperature fluctuations (high: 12.3°C - 22°C; contiguous low: 15.5°C - 25°C; non-contiguous low: 17°C – 26.5°C [24]). 

 Patterns of seasonal change in intensity of infection also differed among site types. At high sites, the mean intensity of infection remained relatively constant across the study period (Figure 4). The pattern at contiguous low sites did not differ significantly from that at high sites after Bonferroni correction, nor did it differ significantly from the pattern at non-contiguous low sites; the pattern of intensity of infection at contiguous low sites was intermediate, as was the pattern of prevalence. However, the patterns of prevalence at contiguous and non-contiguous low sites did differ significantly; in infected individuals, intensity of infection tended to be higher at non-contiguous low sites (Figure 4), except during summer, when the extremely small number of infected individuals, caused by very low prevalence of infection (only one frog was infected at a non-contiguous site in summer), made the estimate of intensity difficult to interpret because of a very wide 95% confidence interval (Figure 4). We have developed three hypotheses for the apparent peak in intensity of infection in summer at contiguous low sites: (i) perhaps only highly infected individuals retained infections, or (ii) perhaps only individuals that selected microhabitats particularly favourable for *Bd* retained infections through the summer. The relatively lower intensities in winter at both types of low elevation sites may reflect the fact that prevalence was increasing at those sites (Figure 3), so that many infected individuals captured in winter were in the early stages of infection. (iii) It is also possible that during winter there was mortality of heavily infected individuals, thus providing a sample of only weakly infected individuals during winter. A similar phenomenon could produce the seasonal changes in prevalence of *Bd* in adult frogs.

Our study suggests that both intraspecific and interspecific reservoir hosts are probably important in the persistence of *Bd* at low elevation sites (both contiguous and non-contiguous), where prevalences in *L. rheocola* fell to near-zero in summer, but increased again the following winter. At contiguous low elevation sites, we have evidence that *L. rheocola* tadpoles had a high prevalence of *Bd* infection (58%), suggesting that they may act as an intraspecific reservoir at these sites [24]. However, the prevalence of infection in tadpoles in non-contiguous sites reached zero in summer [24], indicating that in non-contiguous low sites there must be an interspecific reservoir for *Bd*. Adults of other frog species (e.g. *Litoria wilcoxii*; [15,41]), other higher taxa [42,43], or the environment (water, soil, etc., [6,16,44,45]) may act as reservoirs at non-contiguous sites. We have evidence that terrestrial stages of other frog species in non-contiguous sites were also uninfected in summer (*Nyctimystes dayi, Litoria serrata, Litoria infrafrenata, Litoria leseuri* (complex); A. McNab, unpublished data). It is not clear at present whether *Bd* can persist for long periods as a saprobe or in a resting stage [46]. Recently, it was demonstrated that crayfish harbour *Bd* infections and transmit infection to amphibians in the USA [47]; however, *Bd* infection has not been found in crustaceans in northern Queensland, Australia [48]. It seems most likely that adults or tadpoles of other frog species act as reservoirs for the disease at non-contiguous low sites. The presence of infected tadpoles at contiguous low sites in summer, while they are absent from non-contiguous sites in summer, where air temperatures are similar, strongly suggests that either drift [23] or moderation of water temperatures via flow from cooler upland areas may maintain infections in tadpoles in contiguous low habitats [24]. 

 To our knowledge, our study is the first to examine whether stream connectivity between high and low elevations influences the infection dynamics of *Bd*. We found that the dynamics of both prevalence and intensity of infection differed between sites with and without connectivity to high elevation streams. Our result is consistent with the hypotheses that moderation of water temperature or drift of infectious zoospores (or both) may influence seasonal fluctuations at low sites connected to high elevation streams, as compared to sites not connected to high elevation streams. Our results indicate that reservoir hosts may be necessary for *Bd* to persist in low elevation areas, which are warmer than high elevation sites, possibly allowing more frogs to recover from the disease. Our results also suggest that at low elevation populations that are contiguous with high elevation sites, tadpoles of *L. rheocola* may serve as intraspecific reservoir hosts, since their prevalences of *Bd* infection remained high over summer [24]. At low elevation non-contiguous sites, prevalence of *Bd* in larval *L. rheocola* reached zero over summer, indicating that interspecific reservoirs are necessary to maintain *Bd* at these sites. Fully understanding the factors that determine patterns of infection prevalence in frog populations is an important step towards developing strategies to reduce the impact of chytridiomycosis in natural populations. 
